# Supplementary material for: Identification and Characterization of a Novel CCDC6::CASP7 Gene Rearrangement in an Advanced Colorectal Cancer Patient: A Case Report
Source: Int J Mol Sci. 2024 Nov 26;25(23):12665. doi: 10.3390/ijms252312665 (PMC11641570; doi:10.3390/ijms252312665)
Supplement: Supplementary file 1 [file ijms-25-12665-s001.zip › ijms-3301967-supplementary.pdf]

Variant ID	Type	Gene	Locus	AA Change	Allele Frequency	Genes (Exons)	READ Counts	Gene Isoform	Read Counts Per Million
CCDC6-CASP7.C1C2. Non-Targeted	Fusion	CASP7	Chr10: 61665897-chr10: 115457253			CCDC6(1)-CASP7(2)	115	CCDC6-CASP7.C1C2. Non-Targeted	516
COSM516	snp	KRAS	Chr12:25398284	p.G12C	33.1%				
COSM760	snp	PIK3CA	Chr3:178936082	p.E542K	17.8%				
